# Evaluation of Depth of Invasion and Tumor Thickness as a Prognostic Factor for Early-Stage Oral Squamous Cell Carcinoma: A Retrospective Study

**DOI:** 10.3390/diagnostics12010020

**Published:** 2021-12-23

**Authors:** You-Jung Lee, Tae-Geon Kwon, Jin-Wook Kim, Sung-Tak Lee, Su-Hyung Hong, So-Young Choi

**Affiliations:** 1 Department of Oral & Maxillofacial Surgery, School of Dentistry, Kyungpook National University, 2175, Dalgubeol-daero, Jung-gu, Daegu 41940, Korea; leeyj7544@gmail.com (Y.-J.L.); kwondk@knu.ac.kr (T.-G.K.); vocaleo@knu.ac.kr (J.-W.K.); st0907@knu.ac.kr (S.-T.L.); 2 Department of Microbiology and Immunology, School of Dentistry, Kyungpook National University, 2175, Dalgubeol-daero, Jung-gu, Daegu 41940, Korea; hongsu@knu.ac.kr

**Keywords:** early-stage oral squamous cell carcinoma (OSCC), depth of invasion (DOI), tumor thickness (TT), T category, disease-specific survival (DSS), progression-free survival (PFS)

## Abstract

The aim of this study was to compare the effect of using depth of invasion (DOI) versus tumor thickness (TT) as a prognostic factor for early-stage oral squamous cell carcinoma (OSCC). A total of 57 patients with early-stage OSCC treated surgically from 2009 to 2014 at our institution were reviewed retrospectively. Histopathological measurement of DOI and TT was performed. The validation of DOI and TT as prognostic factors was conducted using a Kaplan–Meier survival analysis. TT had no association with disease-specific survival (DSS) or progression-free survival (PFS) in this cohort; however, increased DOI was significantly associated with decreased DSS but not correlated to decreased PFS. The T category of the 7th edition of AJCC was statistically associated with both DSS and PFS; however, the T category of the 8th edition of the AJCC was only associated with DSS. In this study group, TT could not be used as a prognostic factor, and DOI was not by itself sufficient to predict prognosis for early-stage OSCC. The T category in AJCC 8th Edition cannot be considered the sole prognostic factor for early OSCC, so additional prognostic factors may need to be considered.

## 1. Introduction

The 4th edition of the World Health Organization (WHO) Classification of Tumors of the Head and Neck has adopted the 7th edition of the American Joint Committee on Cancer (AJCC) Staging Manual to evaluate oral cancer patients and their prognosis [1,2]. The tumor (T) category of the 7th edition of the AJCC Staging Manual focuses on the size and extent of the primary tumor. However, the 8th edition of the AJCC Staging Manual, released in 2017, included depth of invasion (DOI) of the primary tumor into the T category for oral cancer. 

Previously, DOI and tumor thickness (TT) have been used interchangeably [3,4]. After the most recent revision of the AJCC Staging Manual was published, the differences between DOI and TT were emphasized, and the precise definitions of each were regarded as important. DOI is measured from the adjacent normal basement membrane to the deepest point of the tumor in millimeters, whereas TT is measured from the highest point of the tumor surface to the deepest point of the tumor in millimeters [5,6,7,8,9,10]. Depending on its morphology, TT can either overvalue or undervalue tumor invasiveness. Therefore, only DOI was taken into account in the 8th edition of the AJCC as tumor invasiveness could predict aggressiveness more precisely [11]. 

The primary aim of this study was to compare the value of using DOI versus TT as a prognostic factor for patients with early-stage oral squamous cell carcinoma (OSCC). We also demonstrated whether the T category from the 8th edition of AJCC can perform as a predictor for the prognosis of early-stage OSCC. 

## 2. Materials and Methods

### 2.1. Study Population

Clinicopathological data were selected retrospectively from patients with early-stage OSCC from 2009 to 2014 at the Department of Oral and Maxillofacial Surgery, Kyungpook National University Hospital, Daegu, South Korea. Data for a total of 105 patients were available, but we only chose those patients who were surgically treated. We applied the following inclusion criteria for early-stage OSCC:

(1) Absence of positive lymph nodes, lymphovascular invasion, or perineural invasion.
(2) Primary OSCC was limited only to soft tissue (absence of bone invasion).
(3) Tumor size greater than 4 cm was excluded as it was considered a more progressed state.

Moreover, patients with positive margins were excluded as it was difficult to confirm the tumor sizes. A total of 57 patients with clinical or pathological T1-2N0 were reviewed in our final analysis. This increased the homogeneity of the patient population, and DOI or TT was evaluated as a sole variable. All subsites of the oral cavity were included. Initially, cancer staging was based on the 7th edition of the AJCC system. 

**Figure 1:**
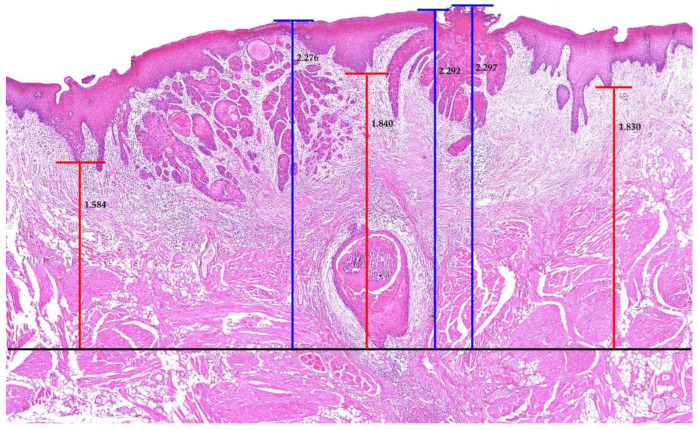
Measurement of depth of invasion (DOI) and tumor thickness (TT), using iSolution Lite software (2003–2015, IMT i-solution Inc., Vancouver, BC, Canada). Measuring was repeated three times for each slide, and the mean value was used as the final value. (×40, Blue line; tumor thickness, Red line; depth of invasion).

Multiple sections of pathology slides were examined with a digital microscope to identify the deepest part of tumor invasion. The histopathological measurement of DOI and TT was conducted using iSolution Lite software (2003–2015, IMT i-solution Inc., Vancouver, BC, Canada). All measurements were repeated three times for each slide, where the average was ultimately used as the final value (Figure 1). A double board-certified pathologist confirmed the results. Patients were divided into three groups for each part: (1) classification according to DOI: group A = DOI ≤ 5 mm, group B = 5 mm < DOI ≤ 10 mm, and group C = DOI > 10 mm; and (2) classification according to TT: group A = TT ≤ 5 mm, group B = 5 mm < TT ≤ 10 mm, and group C = TT > 10 mm.

The collection and use of data for this study were approved by the Institutional Ethics Review Board of the ethics committee at our institution. 

### 2.2. Statistical Analysis

A descriptive analysis was performed for categorical and continuous variables with summary measures. For continuous variables, mean values were reported, whereas percentages were used for categorical variables. The validation of DOI and TT as prognostic factors was visualized using Kaplan–Meier survival curves. Only disease-specific survival (DSS) was calculated as there were no patients who died from other reasons than OSCC in the cohort. The time period for DSS began at the time of the first visit and ended at the time of death. Progression-free survival (PFS), the period between primary treatment and relapse, was also visualized using Kaplan–Meier survival curves to determine recurrence for the patients in this study. The differences in survival rates were compared by the log-rank test. A *p*-value < 0.05 was considered statistically significant. All statistical analyses were performed using SPSS software (version 22.0; SPSS, Chicago, IL, USA).

## 3. Results

### 3.1. Patient Characteristics

A total of 57 patients with early-stage OSCC were included in the study. The clinicopathological characteristics of the population are shown in Table 1. The median age was 59 years (range: 25–85), and the median follow-up period was 4.85 years (range: 0.72–10.50 years). In addition, 61.4% of the patients (*n* = 35) were male, and 49.1% (*n* = 22) had primary oral tongue cancer. The median DOI for all oral subsites was 4.59 mm (range: 0.30–23.20), and the medial TT was 6.95 mm (range: 1.00–21.73). All patients had surgical excision of the primary tumor. The majority of patients (66.7%, *n* = 38) had elective neck dissection (END), but only 15.8% (*n* = 9) received postoperative treatment. Of these patients, 22 had a locoregional recurrence. 

The classification of the pathological T category according to the 7th and 8th editions of the AJCC Staging Manual is shown in Table 1. Based on DOI as a T category modifier, of 35 patients with AJCC 7 T1 stage, 12 were upstaged to T2 and 1 to T3. Of 22 patients with AJCC 7 T2 stage, 8 were upstaged to T3.

### 3.2. Disease-Specific Survival and Progression-Free Survival

**Figure 2:**
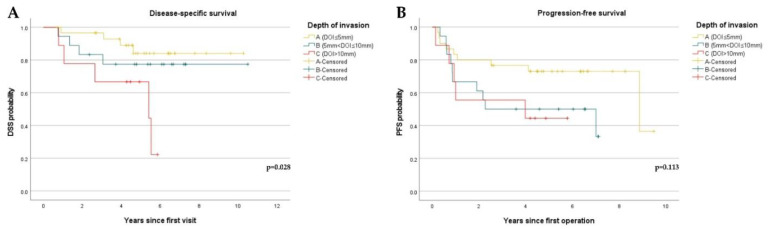
Kaplan–Meier curves showing disease-specific survival (DSS) and progression-free survival (PFS) stratified by depth of invasion (DOI): (**A**) DSS curves stratified by DOI. (A [DOI ≤ 5 mm], *n* = 30, B [5 mm < DOI ≤ 10 mm], *n* = 18, C [DOI > 10 mm], *n* = 9); (**B**) PFS curves stratified by DOI. (A [DOI ≤ 5 mm], *n* = 30, B [5 mm < DOI ≤ 10 mm], *n* = 18, C [DOI > 10 mm], *n* = 9).

A total of 13 patients (22.8%) died. Of them, 9 patients had tumors on the tongue, one had on the gingiva, one had on buccal mucosa and the other had on the mouth floor. Elective neck dissection (END) had not been performed on 4 of them (tongue; 3, mouth floor; 1). All of them died from disease progression with locoregional recurrence, and the median DOI was 9.42 mm. Increasing DOI was associated with decreased DSS as shown in Figure 2A (*p* = 0.028). When comparing groups A, B, and C of DOI with their five-year DSS, group C exhibited a significantly worse five-year DSS (66.7%). However, DOI had no significant association with PFS (*p* = 0.113) among the three groups (Figure 2B).

**Figure 3:**
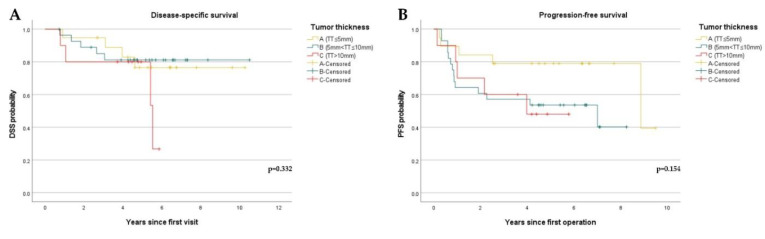
Kaplan–Meier curves showing disease-specific survival (DSS) and progression-free survival (PFS) stratified by tumor thickness (TT): (**A**) DSS curves stratified by TT. (A [TT ≤ 5 mm], *n* = 19, B [5 mm < TT ≤ 10 mm], *n* = 28, C [TT > 10 mm], *n* = 10); (**B**) PFS curves stratified by TT. (A [TT ≤ 5 mm], *n* = 19, B [5 mm < TT ≤ 10 mm], *n* = 28, C [TT > 10 mm], *n* = 10).

The validation of TT was also performed by analyzing DSS and PFS. The median TT of dead patients was 9.14 mm. However, as shown in Figure 3, TT had no significant correlation with either DSS (*p* = 0.332) or PFS (*p* = 0.154).

**Figure 4:**
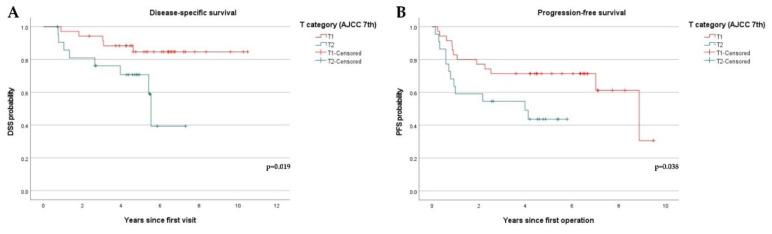
Kaplan–Meier curves showing disease-specific survival (DSS) and progression-free survival (PFS) stratified by the T category of the 7th edition of the AJCC system: (**A**) DSS curves stratified by the T category based on the 7th edition of the AJCC. (T1, *n* = 35, T2, *n* = 22); (**B**) PFS curves stratified by T category based on the 7th edition of AJCC. (T1, *n* = 35, T2, *n* = 22).

**Figure 5:**
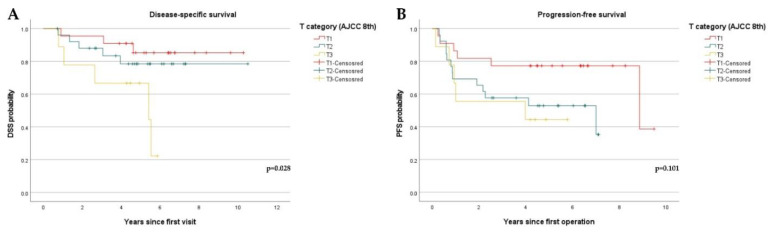
Kaplan–Meier curves showing disease-specific survival (DSS) and progression-free survival (PFS) stratified by T category of the 8th edition of the AJCC system: (**A**) DSS curves stratified by T category (DOI) based on the 8th edition of the AJCC. (T1, *n* = 22, T2, *n* = 26, T3, *n* = 9); (**B**) PFS stratified by T category based on criteria of the 8th edition of the AJCC. (T1, *n* = 22, T2, *n* = 26, T3, *n* = 9).

Based on the 7th edition of the AJCC, an increase in the T category was significantly associated with both decreased DSS (*p* = 0.019) and PFS (*p* = 0.038) (Figure 4). However, based on the 8th edition, an increase in the T category was only associated with decreased DSS (*p* = 0.028) (Figure 5). 

## 4. Discussion

Previously, the terminology for DOI and TT has been used interchangeably [3,4]. However, the precise definition of each term was considered important, and DOI, not tumor thickness (TT), was considered appropriate as a prognostic factor for OSCC [4,9,11,12]. The 8th edition of the AJCC Staging Manual adopted the DOI of the primary tumor into the T category in 2017 to improve prognosis and establish a protocol for the treatment of oral cancer. In this study, we compared DOI and TT as prognostic factors for patients with early-stage OSCC. Moreover, we evaluated whether the T category of the 8th edition of AJCC can be a prognostic factor for early-stage OSCC. 

In this study, we evaluated a unique patient group, early-stage OSCC, to determine whether the T category is a sole prognostic factor while excluding the high-risk pathological features. First, the patients were stratified by the T category of the 7th edition of AJCC. Using the DOI or TT as a T category modifier in the 8th edition of AJCC, the patients were more stratified. A total of 21 patients were upstaged using DOI as a modifier, and 29 patients were upstaged using TT. We attempted to measure DOI and TT according to previous studies [7,11]; however, we had difficulty in determining the precise reference points in the pathology slides and anatomical differences on each oral subsite. Additionally, it was difficult to determine the total size or features of the tumor mass as this study utilized retrospective characteristics. To overcome these limitations and measure DOI and TT more precisely, we referred to the study of Kukreja et al. (2020), who made an effort to not make soft tissue deformation and selected the pathology slides with definite normal adjacent mucosa [13]. In addition, we used a digital microscope and iSolution Lite software (2003–2015, IMT i-solution Inc., Vancouver, BC, Canada) for the measurements. All measurements were repeated three times, and the final value was determined by a mean value. A double board-certified pathologist reviewed the results. 

Unlike the previous studies [3,7,14], we concluded that TT had no efficacy as a prognostic factor for patients with early-stage OSCC. This may have resulted from confusion on the use of DOI and TT in previous studies or an insufficient number of subjects. Although the study of Faisal et al. (2018) reported that DOI is a sole prognostic factor for patients with early-stage OSCC and verified that DOI is associated with DSS and locoregional recurrence [8], the present study concluded that increasing DOI is only associated with decreasing DSS and does not influence the time-to-relapse or recurrence rate. Our results are consistent with that of Kozak et al. (2018) and Tam et al. (2019) and suggested the possibility of a cofactor affecting recurrence time regardless of DOI [10,15]. Other studies [6,16,17] described adverse pathological factors, such as deep positive margin, perineural invasion, and worst pattern of invasion, as cofactors. However, this study was limited by patient selection, and it was not possible to identify the correlation between prognosis and these factors. 

Kano et al. (2018) suggested that the possibility of nodal metastasis in patients with T1-2N0 was not different for the criteria in the 7th and 8th editions [18]. Therefore, the same management is needed for patients with N0 disease. According to the results of this study, it was demonstrated that the T category of the 7th edition of the AJCC was more robust than that of the 8th edition. There was no significant evidence that the T category of the 8th edition is a better prognostic factor compared with the 7th edition.

Piazza et al. (2019) and Bjerkli et al. (2020) suggested that the 8th edition of the AJCC staging system improved the prognostic value of the T category than the previous one in early-stage oral tongue SCC [19,20]. Contrary to Piazza et al. (2019) and Bjerkli et al. (2020), our results are more relevant to Kano et al. (2018) As this study included a relatively small number of patients and all oral cavity subsites, additional studies using a larger patient population are needed.

This study was performed using all oral cavity subsites to reach a general conclusion. The previous studies also included all subsites, such as tongue, mouth floor, retromolar trigone, gingiva, buccal mucosa, hard palate, and even lip [7,15]. However, many studies performed analyses only on oral tongue cancer [6,8,10,12,14,16,18] or the buccal cheek [5]. As the tongue is the most common subsite of oral squamous cell carcinoma (OSCC) and has a higher risk of cervical lymph node metastasis than other subsites with regard to its abundant lymphatics and mouth floor [8], lots of studies analyzed the pathology of oral tongue squamous cell carcinoma (OTSCC). As shown in the study by Brockhoff et al. (2017), each oral subsite has different considerations, and the cut-off point for each treatment plan may be different [21]. A sufficient population size for each subsite is needed to analyze the site-specific characteristics and design a more specific treatment plan.

There are several limitations in this study including its relatively small cohort size, retrospective nature, and possible bias for sample selection. Because the study was conducted at a single institution, the results may not be suitable for generalization to a large population. This study represented a retrospective cohort analysis, and therefore, the postoperative adjuvant treatment was performed according to the pathological characteristics of the disease rather than being randomly selected. In addition, 19 patients did not undergo END. Although lymph node metastasis was not detected during the preoperative imaging diagnosis, it is not possible to identify node metastasis of the patients who did not have the END. This could result in biased sample selection and analysis. In addition, we excluded the patients with more progressive disease, and these results may underestimate and exclude the true prognostic factors for OSCC.

## 5. Conclusions

Based on the analysis of this study cohort, TT was not a significant prognostic factor for early-stage OSCC. Depth of invasion was associated with the DSS rate of early-stage OSCC but was not associated with time to recur. This study showed that DOI is not sufficient as a prognostic factor in patients with early-stage OSCC, so consideration of additional prognostic factors in patients with early-stage OSCC may be necessary.

## Figures and Tables

**Table 1:** Clinicopathological characteristics of the 57 patients in the study.

Variables		N (%)
Age (yrs)		59 (25–85)
Sex	Male	35 (61.4)
	Female	22 (38.6)
Oral subsites	Tongue	28 (49.1)
	Palate	3 (5.3)
	Retromolar trigone	2 (3.5)
	Gingiva	11 (19.3)
	Buccal mucosa	9 (15.8)
	Mouth floor	4 (7.0)
pT (AJCC 7th)	pT1	35 (61.4)
	pT2	22 (38.6)
pT (AJCC 8th/DOI)	pT1	22 (38.6)
	pT2	26 (45.6)
	pT3	9 (15.8)
pT (AJCC 8th/TT)	pT1	14 (24.6)
	pT2	35 (56.1)
	pT3	11 (19.3)
Tumor thickness (mm) *		6.95 (1.00–21.73)
Depth of invasion (mm) *		4.59 (0.30–23.20)
Neck dissection	No ND	19 (33.3)
	Elective ND	38 (66.7)
Overall treatment	Surgery only	48 (84.2)
	Surgery + adjuvant treatment	9 (15.8)
Recurrence		22 (38.6)

* Median (range) given for continuous variables. In this study, pN classification of entire cases was the same in the 7th and 8th editions of the AJCC manuals.

## Data Availability

The data presented in this study are available in the article.

## References

[1] Kennedy R.A. (2018). WHO is in and WHO is out of the mouth, salivary glands, and jaws sections of the 4th edition of the WHO classification of head and neck tumours. Br. J. Oral Maxillofac. Surg..

[2] Müller S. (2017). Update from the 4th edition of the World Health Organization of head and neck tumours: Tumours of the oral cavity and mobile tongue. Head Neck Pathol..

[3] Aslam F., Atique M., Aslam M., Sarfraz T., Ayaz B.A.B., Alamgir W. (2012). Relation of tumour thickness with lymph node metastasis in oral squamous cell carcinoma. Pak. Armed Forces Med. J..

[4] Pentenero M., Gandolfo S., Carrozzo M. (2005). Importance of tumor thickness and depth of invasion in nodal involvement and prognosis of oral squamous cell carcinoma: A review of the literature. Head Neck J. Sci. Spec. Head Neck.

[5] Ahmed S.Q., Junaid M., Awan S., Choudhary M.M., Kazi M., Masoom A., Khan H.U. (2017). Relationship of tumor thickness with neck node metastasis in buccal squamous cell carcinoma: An experience at a tertiary care hospital. Int. Arch. Otorhinolaryngol..

[6] Berdugo J., Thompson L.D., Purgina B., Sturgis C.D., Tuluc M., Seethala R., Chiosea S.I. (2019). Measuring depth of invasion in early squamous cell carcinoma of the oral tongue: Positive deep margin, extratumoral perineural invasion, and other challenges. Head Neck Pathol..

[7] Dirven R., Ebrahimi A., Moeckelmann N., Palme C.E., Gupta R., Clark J. (2017). Tumor thickness versus depth of invasion–Analysis of the 8th edition American Joint Committee on Cancer Staging for oral cancer. Oral Oncol..

[8] Faisal M., Abu Bakar M., Sarwar A., Adeel M., Batool F., Malik K.I., Jamshed A., Hussain R. (2018). Depth of invasion (DOI) as a predictor of cervical nodal metastasis and local recurrence in early stage squamous cell carcinoma of oral tongue (ESSCOT). PLoS ONE.

[9] Liu B., Amaratunga R., Veness M., Wong E., Abdul-Razak M., Coleman H., Gebski V., Sundaresan P. (2020). Tumor depth of invasion versus tumor thickness in guiding regional nodal treatment in early oral tongue squamous cell carcinoma. Oral Surg. Oral Med. Oral Pathol. Oral Radiol..

[10] Tam S., Amit M., Zafereo M., Bell D., Weber R.S. (2019). Depth of invasion as a predictor of nodal disease and survival in patients with oral tongue squamous cell carcinoma. Head Neck.

[11] Lydiatt W.M., Patel S.G., O’Sullivan B., Brandwein M.S., Ridge J.A., Migliaccim J.C., Loomis A.M., Shah J.P. (2017). Head and neck cancers—Major changes in the American Joint Committee on cancer eighth edition cancer staging manual. CA A Cancer J. Clin..

[12] Tan W.J., Chia C.S., Tan H.K., Soo K.C., Iyer N.G. (2012). Prognostic significance of invasion depth in oral tongue squamous cell carcinoma. ORL.

[13] Kukreja P., Parekh D., Roy P. (2020). Practical challenges in measurement of depth of invasion in oral squamous cell carcinoma: Pictographical documentation to improve consistency of reporting per the AJCC 8th edition recommendations. Head Neck Pathol..

[14] Gonzalez-Moles M.A., Esteban F., Rodriguez-Archilla A., Ruiz-Avila I., Gonzalez-Moles S. (2002). Importance of tumour thickness measurement in prognosis of tongue cancer. Oral Oncol..

[15] Kozak M.M., Shah J., Chen M., Schaberg K., von Eyben R., Chen J.J., Bui T., Kong C., Kaplan M., Divi V. (2019). Depth of invasion alone as a prognostic factor in low‐risk early‐stage oral cavity carcinoma. Laryngoscope.

[16] Almangush A., Bello I.O., Coletta R.D., Mäkitie A.A., Mäkinen L.K., Kauppila J.H., Pukkila M., Hagström J., Laranne J., Soini Y. (2015). For early-stage oral tongue cancer, depth of invasion and worst pattern of invasion are the strongest pathological predictors for locoregional recurrence and mortality. Virchows Arch..

[17] Larson A.R., Kemmer J., Formeister E., El-Sayed I., Ha P., George J., Ryan W., Chan E., Heaton C. (2020). Beyond Depth of Invasion: Adverse Pathologic Tumor Features in Early Oral Tongue Squamous Cell Carcinoma. Laryngoscope.

[18] Kano S., Sakashita T., Tsushima N., Mizumachi T., Nakazono A., Suzuki T., Yasukawa S., Homma A. (2018). Validation of the 8th edition of the AJCC/UICC TNM staging system for tongue squamous cell carcinoma. Int. J. Clin. Oncol..

[19] Piazza C., Bresciani L., Giannini L. (2019). Depth of invasion for prognostic stratification in oral cavity cancer: Do we need further validation?. Ann. Transl. Med..

[20] Bjerkli I.H., Laurvik H., Nginamau E.S., Søland T.M., Costea D., Hov H., Uhlin-Hansen L., Hadler-Olsen E., Steigen S.E. (2020). Tumor budding score predicts lymph node status in oral tongue squamous cell carcinoma and should be included in the pathology report. PLoS ONE.

[21] Brockhoff H.C., Kim R.Y., Braun T.M., Skouteris C., Helman J.I., Ward B.B. (2017). Correlating the depth of invasion at specific anatomic locations with the risk for regional metastatic disease to lymph nodes in the neck for oral squamous cell carcinoma. Head Neck.

